# Comparison of ADM and Connective Tissue Graft as the Membrane in Class II Furcation Defect Regeneration: A Randomized Clinical Trial

**DOI:** 10.5681/joddd.2014.018

**Published:** 2014-06-11

**Authors:** Vahid Esfahanian, Shirin Farhad, Mehrnaz Sadighi Shamami

**Affiliations:** ^1^Assistant Professor, Department of Periodontology, Faculty of Dentistry, Islamic Azad University Khorasgan (Isfahan) Branch, Isfahan, Iran; ^2^Assistant Professor, Faculty of Dentistry, Tabriz University of Medical Science, Tabriz, Iran

**Keywords:** Allograft, connective tissue, DFDBA, furcation defects, guided tissue regeneration

## Abstract

***Background and aims.*** Furcally-involved teeth present unique challenges to the success of periodontal therapy and influence treatment outcomes. This study aimed to assess to compare use of ADM and connective tissue membrane in class II furcation defect regeneration.

***Materials and methods.*** 10 patient with 2 bilaterally class II furcation defects in first and/or second maxilla or man-dibular molar without interproximal furcation involvement, were selected. Four weeks after initial phase of treatment, before and thorough the surgery pocket depth (PD), clinical attachment level to stent (CAL-S), free gingival margin to stent(FGM-S) , crestal bone to stent (Crest-S), horizontal defect depth to stent (HDD-S) and vertical defect depth to stent (VDD-S) and crestal bone to defect depth measured from stent margin. Thereafter, one side randomly treated using connective tissue and DFDBA (study group) and opposite side received ADM and DFDBA (control group). After 6 months, soft and hard tissue parameters measured again in re-entry.

***Results.*** Both groups presented improvements after therapies (P & 0.05). No inter-group differences were seen in PD re-duction (P = 0.275), CAL gain (P = 0.156), free gingival margin (P = 0.146), crest of the bone (P = 0.248), reduction in horizontal defects depth (P = 0.139) and reduction in vertical defects depth (P = 0.149).

***Conclusion.*** Both treatments modalities have potential of regeneration without any adverse effect on healing process. Connective tissue grafts did not have significant higher bone fill compared to that of ADM.

## Introduction

Furcation-involved teeth present unique challenges to the success of periodontal therapy and influence treatment outcomes.^1,2^ An ultimate goal of periodontal treatment is not only to prevent the progression of periodontal disease but also to regenerate lost periodontal tissue.

A variety of materials and techniques including autogenous bone graft,^3^bone substitutes,^4^guided tissue regeneration (GTR)^5^or a combination of these have been used in regeneration of furcation defects.^6^ Guided tissue regeneration uses either a resorbable or a non-resorbable barrier membrane to prevent the migration of epithelial cells, bone and gingival tissues to the wound area and will also provide an opportunity for accumulation of cells in periodontal fibers.^7-10^ However, according to the literature, there is no difference between resorbable and non-resorbable membranes in terms of treatment outcomes.^11-13^Because of higher cost, need for a second surgery for membrane removal, complexity and bacterial accumulation of non-resorbable membranes, absorbable membranes are preferable.^14,15^ Subepithelial connective tissue cells contain mesenchymal cells and have the osteogenic, chondrogenic and osteoblastic capacities.^15,16-19^ These cells are also capable of modulating the immune system.^20^ On the other hand, gingival tissue is a richer source of mesenchymal stem cells in comparison with the bone marrow.^21^Using palatal autogenous connective tissue graft as a barrier membrane in regeneration of furcation defects is regarded as a proper treatment with advantages like lower cost, availability, and adaptability.^16,17,22^

Acellular dermal matrix (ADM), material obtained from human skin, has been used as a substitute for palatal connective tissue to increase the width of keratinized tissue around teeth or implants,^24,25^ for the treatment of alveolar ridge deformities,^26^ and for root coverage procedures.^27-30^ Some clinical studies^31-36^ used ADM as a membrane for guided bone regeneration in edentulous ridges and in association with immediate implants, suggesting that this material may be able to act as a barrier.

Bone grafts are used in treatment of alveolar bone lesions because of their osteoconductive or osteoinductive properties and in maintaining the space under the membrane and preventing it from collapsing into the defect.^14-17^ They also facilitate wound stability, providing space to enable the regeneration process.^8^ Therefore, it is recommended to use bone graft for furcation defects treatment. To date, there are no studies to compare the barrier function of connective tissue grafts and acellular dermal matrix in GTR.^14,16,17,18,22,23^This study aimed to assess to compare use of ADM and connective tissue membrane in class II furcation defect regeneration.

## Materials and Methods

This study was a single-blinded randomized clinical trial with split mouth design (registration number IRCT201254569833N1 in Iranian Registry of Clinical Trials). 10 patients with moderate to severe periodontitis attending the Department of Periodontics, Islamic Azad University Khorasegan (Isfahan) Branch, for periodontal treatment were enrolled in this study. Inclusion criteria: Patients with at least one paired vital or non-vital (with appropriate root canal therapy) teeth with class II furcation defect and plaque index of 25% or less according to O’Leary plaque index prior to surgery.^40^ Exclusion criteria: Patients with any systemic disease or conditions, pregnant or breast feeder, patients with other types of chronic periodontitis or tooth mobility, patients with history of periodontal surgery in the last six months, and patients under any medications. The study design as well as any risks or discomforts were explained to the patients and written informed consents were taken.

After oral health instruction, scaling and root planning was performed in two sessions by means of ultrasonic devices with one week interval. After two months evaluation of phase one treatment, the selected sites underwent periodontal surgery.

**Figure1.**
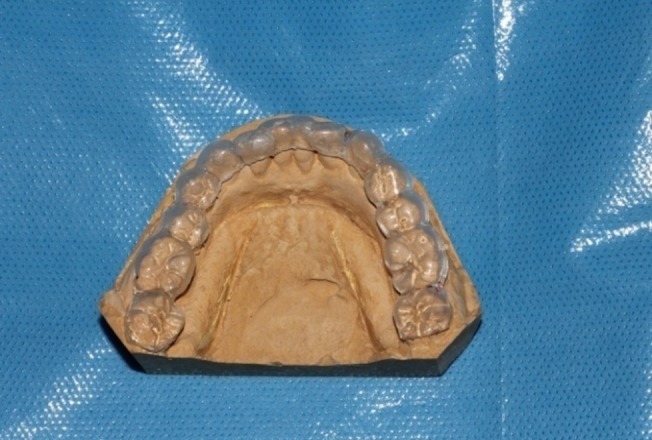
Prefabricated acrylic stent.

Acrylic stents were fabricated using a dental cast of each patient. To fabricate the acrylic stents, one third of the occlusal portion of the teeth with intra-osseous lesions and at least one tooth in mesial and distal aspect of the selected site was covered by acrylic resin, except in cases that the offended tooth was the most distally positioned tooth in the arch (Figure 1).

Clinical parameters including probing pocket depth, clinical attachment level, and gingival margin position were recorded using the acrylic stent and a UNC-15 periodontal probe. To ensure perfect alignment of the acrylic index in place, the method of determining the distance to CEJ was used in the acrylic structure. Also for reproducible and reliable soft and hard tissue evaluations, guide slots were created in the stent structure. These tracks on preoperative casts were prepared so that the probe could be placed parallel with the long axis of the tooth. According to the entry angle of the probe, the groove slot was produced in acrylic structures. This groove was a guide to determine the filling of the lesions and also to record the changes after surgery.

The measured parameters included pocket depth (PD), clinical attachment level to stent (CAL-S), free gingival margin to stent (FGM-S), free gingival margin, crestal bone to stent (Crest-S), horizontal defect depth to stent (HDD-S), and vertical defect depth to stent (VDD-S).

###  First Surgery

**Figure 2.**
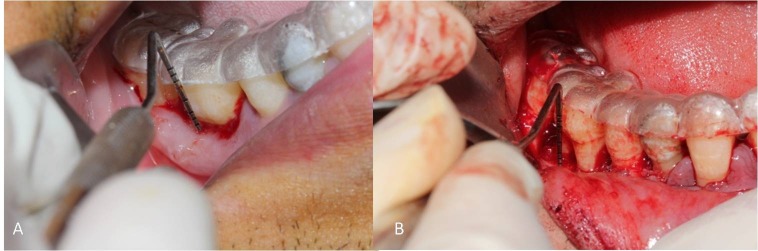
Soft tissue (A) and hard tissue (B) measurement before surgery.

After soft tissue measurements prior to surgery local anesthesia was provided with 2% lidocaine containing 1/80,000 epinephrine (Darou Pakhsh, Tehran, Iran). Sulcular incision was made by scalpel No. 15 in one tooth, mesial and distal of the treatment area, in the buccal and lingual aspects. A mucoperiosteal flap was elevated 3 mm beyond the margins of the furcation defect. After complete debridement of granulation tissues from the defect walls and inner surfaces of flap, root surfaces were planned. The acrylic stent was again placed in the area and hard tissue parameters including crestal bone to stent (Crest-S), horizontal defect depth to stent (HDD-S), and vertical defect depth to stent (VDD-S) were measured by means of an UNC-15 periodontal probe (Figure 2). To obtain palatal connective tissue graft, after anesthesia with 2% lidocaine containing 1/80000 epinephrine, a horizontal incision with 3-mm distance from the palatal gingival margin was made by a scalpel blade No. 15 in the site of the first molar to the first premolar. Two vertical incisions from the terminal points of the horizontal incision were made toward the midline of the palate. A thickness of 1-1.5 mm of the underlying connective tissue was dissected by sharp dissection and was stored in normal saline soaked gauze.

**Figure 3.**
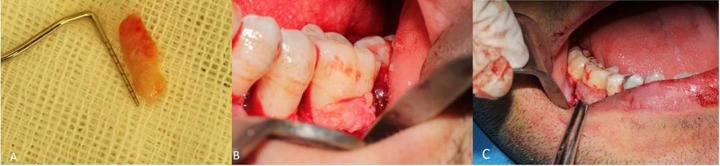
Connective tissue graft (A) adaptation in furcation site defects (B). ADM adaptation in furcation site defects (C).

The palatal flap was sutured and covered with periodontal dressing. In both groups, DFDBA (0.5; 150-2000 µm Cortical Cancellous Powder, Tissue Regeneration Corporation, Kish Island, Iran) was used as the graft material. The granules were slightly formed by a sterile spatula. Connective tissue graft one side and ADM (1×1cm; Tissue Regeneration Corporation, Kish Island, Iran) on opposite site were shaped to cover the defect without tension and in a way to be secured on bony margins (Figure 3). Horizontal cross mattress suture was used to stabilize the connective tissue graft and ADM in the desired position and the coronal edges of the flap were sutured using the 0-4 silk suture using interrupted technique.

###  Post-surgery Phase

After surgery, 500 mg amoxicillin three times a day for a week, 400 mg Ibuprofen for pain control, and 0.2% chlorhexidine mouthrinse twice a day for 2 weeks were prescribed. Patients were visited 14 days later for suture removal. During the first month, patients were visited every two weeks and each time the entire mouth was examined and professional prophylaxis was performed. After the first month patients were visited monthly for six months. In all these visits, hygiene instruction and debridement were performed if necessary.

###  Second Surgery (Re-entry)

Prior to re-entry surgery, acrylic stent was placed by the same person and all of the soft tissue parameters were recorded. Lidocaine 2% containing 1/80000 epinephrine was used for local anesthesia and sulcular incision was made by No. 15 blade in one tooth mesial and distal to the area. A mucoperiosteal flap was retracted. Acrylic index was placed again for hard tissue measurements (Figure 2). In addition, we used an endodontic file with a rubber stop to measure the horizontal defects change parallel to the stent. The flaps were sutured with 0-4 silk. Patients were visited to remove the sutures after 10 days. Data were analyzed by independent *t*-test to compare the results between the groups. Also to compare the results in each group, paired *t*-tests (α = 0.05) was used. P < 0.05 was considered statistically significant.

## Results

**Figure 4.**
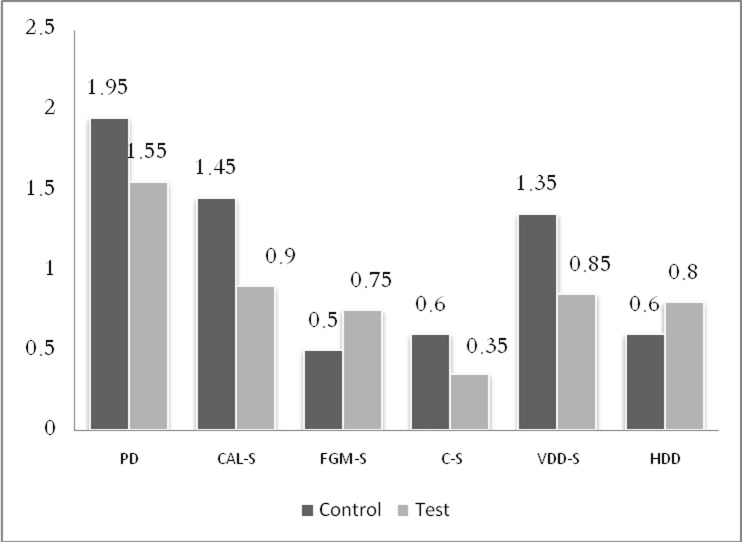
Mean differences in the evaluated parameters (in millimeters) at baseline and after 6 months in the test and control groups.

Ten individuals including eight men and two women with a mean age of 45.2 ± 5.8 years enrolled in this study. The results of the study are summarized in Table 1 and Figure 4.

**Table 1:** The evaluated parameters at baseline and after 6 months in the test and control groups

Clinical parameters	Test group			Control group			P value
	Baseline	6 Months	Changes	Baseline	6 Months	Changes	
PPD^1^	4.75±1.2	2.8±0.8	1.95	4.65±0.5	3.1±0.7	1.55	0.275
CAL^2^	10.8±1.7	9.35±2.5	1.45	10. 9±1.9	10±2.4	0.9	0.156
FGM-S^3^	6.05±2.1	6.55±1.9	0.5	6.25±2.2	6.9±1.3	0.75	0.146
Crest-S^4^	10.65±1.5	11.05±1.6	0.6	10.8±1.8	11.15±1.2	0.35	0.148
Vertical Defect Depth-S^5^	12.56±2.7	11.4±1.77	1.25	12.85±2.3	12±2.2	0.85	0.194
Horizontal Defect Depth-S^6^	2.0±0.7	1.35±0.4	0.6	2.3±0.6	1.5±0.2	0.8	0.139
P < 0.05 was considered statistically significant							
^1^Probing Pocket Depth (changes show depth reduction)							
^2^Clinical Attachment Level (changes show clinical attachment gain)							
^3^Free Gingival Margin to Acrylic Stent (changes show gingival recession)							
^4^Alveolar Bone Crest to Acrylic Stent (indicates crestal recession)							
^5^Vertical Defect Depth to Acrylic Stent (changes show defect fill in vertical direction)							
^6^Horizontal Defect Depth to Acrylic Stent (changes show defect fill in horizontal direction)							

All parameter changes were statistically significant in each group after 6 month. However, mean changes in parameters were not statistically significant between test and control groups. After six month the following were recorded for the test and control groups, in the respective order: the mean changes of PD 1.95 and 1.55 (P = 0.275), CAL gain 1.45 and 0.9 (P = 0.156), free gingival margin 0.5 and 0.75 (P = 0.146), crest of the bone 0.6 and 0.35 (P = 0.248), reduction in horizontal defects depth 1.25 (P = 0.139) and 0.85, and reduction in vertical defects depth 0.6 and 0.8 (P = 0.149).

## Discussion

Acting as a tolerated biological barrier membrane that prevents the epithelial cells from proliferation into the lesion site, palatal connective tissue shows no proliferation into the lesion in the presence of a support underneath.^20^ Connective tissue grafts have the ability to stimulate osteogensis in the periodontally diseased area and can be considered as a good alternative in regenerative modalities.^25^

On the other hand, it has been reported connective tissue contains mesenchymal cells that can result in even superior osteogenicity, chondrogenicity, and osteoblastic capacities.^20,21^

Based on the results of the present study, the mean pocket depth reductions in the test group (connective tissue graft and DFDBA) and control group (ADM and DFDBA) were 1.95 mm and 1.55 mm, respectively. Moghaddas and Zamani^17^ found 3.5 mm probing pocket reduction using palatal connective tissue graft, which is in the approximate range of our findings. Minor differences in results could be due to differences in the type and the details of the treatments rendered.

In addition, attachment gain in the test and control groups were 1.45 mm and 0.9 mm, respectively, indicating that both methods were effective in gaining attachment. Attachment gain improvement values in similar studies were 1.5 mm,^15^ 2.1 mm,^17^ 3.2 mm,^14^ 2.8 mm,^36^ and 2.2 mm,^16^ which are all within a narrow range.

The results of the present study demonstrated almost no change in the position of the gingival margin (gingival recession) between the test (connective tissue graft and DFDBA) and control groups (ADM and DFDBA; 0.5 mm and 0.75 mm, respectively; P = 0.146). This finding was also comparable to studies of Moghaddas and Zamani (0.9 mm),^15^ Moghaddas and Ghasemi (0.5 mm),^42^ Kwan et al(0.3 mm),^16^ and Esfahanian et al (0.07 mm).^41^ The minimal gingival recession that was observed in this study is regarded as a great advantage for the use of connective tissue, since one reason for using regenerative methods is esthetic considerations. Thus, prevention of gingival recession, especially in the anterior region, will provide greater patient satisfaction. In studies that have compared collagen membranes and connective tissue in regeneration of furcation defects, gingival margin positions have significantly lower rates of recession in connective tissue groups, which is probably a result of simultaneous soft tissue augmentation provided by the connective tissue.^15,33,39^

Minor differences among the present results and those of other studies can be justified by initial defect depth (the deeper the defect, the more gain in attachment and bone fill) and differences in the types of materials used in the surgical treatment.^8^ In this study, it was shown that palatal connective tissue can prevent recession. Both groups had no significant differences in terms of gingival recession after GTR. The studies of Paolantonio et al,^33^Moghaddas et al,^15^ and Westfelt et al.^39^ on comparison of collagen and palatal connective tissue membranes have shown no significant differences regarding treatment efficacy.

In the present study, no differences were seen utilizing connective tissue graft and ADM, which may indicate the connective tissue itself has the ability to promote the regeneration process equal to other membranes such as ADM.

## Conclusion

Both the connective tissue graft and ADM can significantly improve clinical parameters in regeneration of furcation defects with no significant difference.

## References

[1] Ramfjord SP, Caffesse RG, Morrison EC, Hill RW, Kerry GJ, Appleberry EA (1987). 4 modalities of periodontal treatment compared over 5 years. J Clin Periodontol.

[2] Hirschfeld L, Wesserman B (1978). A Long-term survey of tooth Joss in 600 treated periodontal patients. J Periodontal.

[3] Quintero G, Mellonig JT, Cambill VM, Pelleu GB (1982). A six month clinical evaluation of decalcified freeze-dried bone allografts in periodontal osseous defects. J Periodontol.

[4] Kenney EB, Lekovic V, Sa Ferreira JC, Han T, Dimitrijevic B, Carranza FA Jr (1986). Bone formation within porous hydroxylapatite implants in human periodontal defects. J Periodontol.

[5] Becker W, Becker B, Prichard J, Caffesse R, Rosenberg E, Gian-Grasso J (1987). Root isolation for new attachment proceduresA surgical and suturing method: Three case reports. J Periodontol.

[6] Cortellini P, Bowers GM (1995). Periodontal regeneration of intrabony defects: and evidence-based treatment approach. Int J Perio Rest Dent.

[7] Sculean A, Donos N, Chiantella GC (1999). GTR with bioabsorbable membranes in the treatment of intrabony defects: A clinical and histological study. Int J Perio Rest Dent.

[8] Taktakis CC, Promsudthi A (1999). Devices for periodontal regeneration. Periodontol 2000.

[9] Cortellini P, Tonneti S (2000). Focus on intrabony defect: GTR. Periodontol2000.

[10] Cortellini P, Tonneti S (2005). Clinical performance of a regeneration strategy for intrabony defects: scientific evidence and clinical experience. J Periodontol.

[11] Gottlow J (1993). Guided tissue regeneration using bioresorbable and non-resorbable devices: Initial healing and long-term results. J Periodontol.

[12] Chen CC, Wang HL, Smith F, Glickman GN, Shyr Y, O’Neal RB (1995). Evaluation of collagen membrane with and without bone grafts in treating periodontal intrabony defects. J Periodontol.

[13] Christgau M, Schmalz G, Reich E, Wenzel A (1995). Clinical and radiographical split-mouth-study on resorbable and nonresorbable GTR-membranes. J Clin Periodontol.

[14] Cortellini P, PiniPrato G, Tonetti MS (1996). Periodontal regeneration of human intrabony defects with bioresorbable membranesA controlled clinical trial. J Periodontol.

[15] Moghaddas H, Soltani L, Moghaddas O (2010). Efficacy of palatal connective tissue as a membrane in treatment of intrabony defects. J Periodontol Implant Dent.

[16] Kwan S, Lekovic V, Camargo P, Kenny E (1998). The use of autogenous periosteal grafts as barriers for the treatment of intrabony defects in human. J Periodontol.

[17] Moghaddas H, Zamani AR (2000). The use of palatal connective tissue as a barrier in Treatment of intrabony defects. J Iranian Dental Association.

[18] Lafzi A, Shirmohammadi A, Faramarzi M, Jabali S, Shayan A. Clinical comparison of autogenous bone graft with and without plasma rich in growth factors in the treatment of grade II furcation involvement of mandibular molars. J Dent Res Dent Clin Dent Prospects 2013;722-9. 10.5681/joddd.2013.004 PMC3593201 23486928

[19] Fournier BP, Ferre FC, Couty L, Lataillade JJ, Gourven M, Naveau A (2010). Multipotent progenitor cells in gingival connective tissue. Tissue Eng Part A.

[20] Mitrano TI, Grob MS, Carrion F, Nova-Lamperti E, Luz PA, Fierro FS (2010). Culture and characterization of mesenchymal cells from human gingival tissue. J Periodontol.

[21] Tomar GB, Srivastava RK, Gupta N, Barhanpurker AP, Pote ST (2010). Human gingiva-derived mesenchymal stem cells are superior to bone marrow derived mesenchymal stem cells for cell therapy in regenerative medicine. Biochem Biophys Res Commun.

[22] Lekovic V, Kenney EB, Carranza FA, Martignoni M (1991). Use of autogenous periosteal grafts as a barrier for treatment of grade II furcation involvement in lower molars. J Periodontol.

[23] Paolantonio M, Femminella B, Coppolino E, Sammartino G, D'Arcangelo C, Perfetti G (2010). Autogenous periosteal barrier membranes and bone grafts in the treatment of periodontal intrabony defects of single-rooted teeth: a 12-month reentry randomized controlled clinical trial. J Periodontol.

[24] Wei PC, Laurell L, Geivelis M, Lingen MW, Maddalozzo D (2000). Acellular dermal matrix allografts to achieve increased attached gingivaPart 1A clinical study. J Periodontol.

[25] Harris RJ (2001). Clinical evaluation of 3 techniques to augment keratinized tissue without root coverage. J Periodontol.

[26] Batista EL Jr, Batista FC, Novaes AB Jr (2001). Management of soft tissue ridge deformities with acellular dermal matrixClinical approach and outcome after 6 months of treatment. J Periodontol.

[27] Aichelmann-Reidy ME, Yukna RA, Evans GH, Nasr HF, Mayer ET (2001). Clinical evaluation of acellular allograft dermis for the treatment of human gingival recession. J Periodontol.

[28] Novaes AB Jr, Grisi DC, Molina GO, Souza SLS, Taba M Jr, Grisi MFM (2001). Comparative 6- month clinical study of a subepithelial connective tissue graft and acellular dermal matrix for the treatment of gingival recession. J Periodontol.

[29] Paolantonio M, Dolci M, Esposito P, D'Archivio D, Lisanti L, Di Luccio A (2002). Subpedicle acellular dermal matrix graft and autogenous connective tissue graft in the treatment of gingival recessions: a comparative 1-year clinical study. J Periodontol.

[30] Barros RRM, Novaes AB Jr, Grisi MFM, Souza SLS, Taba M Jr, Palioto DB (2004). A 6-month comparative clinical study of a conventional and a new surgical approach for root coverage with acellular dermal matrix. J Periodontol.

[31] Novaes AB Jr, Souza SLS (2001). Acellular dermal matrix graft as a membrane for guided bone regeneration: A case report. Implant Dent.

[32] Novaes AB Jr, Papalexiou V, Luczyszyn SM, Muglia VA, Souza SLS, Taba M Jr (2002). Immediate implant in extraction socket with acellular dermal matrix graft and bioactive glass: A case report. Implant Dent.

[33] Luczyszyn SM, Papalexiou V, Novaes AB Jr, Grisi MFM, Souza SLS, Taba M Jr (2005). Acellular dermal matrix and hydroxyapatite in prevention of ridge deformities after tooth extraction. Implant Dent.

[34] Fowler EB, Breault LG, Rebitski G (2000). Ridge preservation utilizing an acellular dermal allograft and demineralized freeze-dried bone allograft: Part IA report of 2 cases. J Periodontol.

[35] Fowler EB, Breault LG, Rebitski G (2000). Ridge preservation utilizing an acellular dermal allograft and demineralized freeze-dried bone allograft: Part IIImmediate endosseous implant placement. J Periodontol.

[36] Griffin TJ, Cheung WS, Hirayama H (2004). Hard and soft tissue augmentation in implant therapy using acellular dermal matrix. Int J Periodontics Restorative Dent.

[37] MoghaddasH MoghaddasH, Kadkhodazadeh M, Pezeshkfar A (2012). Comparison of the Palatal Connective Tissue Graft as a Membrane with Collagen Membrane in Combination with Bio-Oss and PRGF for Treatment of Intrabony Defects: A Randomized Clinical Trial. Journal of Dental School, ShahidBeheshtyUnivercity of Medical Science.

[38] Hanna R, TrejoPM TrejoPM, WeltmanRL WeltmanRL (2004). Treatment of intrabony defects with bovine derived xenograft alone and in combination with platelet rich plasma:a randomized clinical trial. J Periodontol.

[39] Westfelt E, Nyman S, Socransky S, Lindhe J (1983). Significance of frequency of professional tooth cleaning for healing following periodontal surgery. J Clin Periodontol.

[40] O’Leary TJ, Drake RB, Naylor JE (1972). The plaque control record. J Periodontol.

[41] Esfahanian V, Moghaddas H, Moghaddas O (2012). Efficacy of connective tissue graft as a membrane and ABBM with and without PRP in treatment of intrabony defects. Journal of Isfahan Dental School.

[42] Moghaddas H, Ghasemi N (1997). Efficacy of connective tissue graft as a membrane with and without hydroxyapatite in treatment of vertical bony defects. Beheshti Univ Dent J.

